# Supplementary material for: Attenuation of Ventilation-Enhanced Epithelial–Mesenchymal Transition through the Phosphoinositide 3-Kinase-γ in a Murine Bleomycin-Induced Acute Lung Injury Model
Source: Int J Mol Sci. 2023 Mar 14;24(6):5538. doi: 10.3390/ijms24065538 (PMC10053679; doi:10.3390/ijms24065538)
Supplement: Supplementary file 1 [file ijms-24-05538-s001.zip › ijms-2190921-supplementary.pdf]

The following data are supplementary material.
Bronchoalveolar lavage fluid neutrophil counts were examined to quantitate neutrophils, the main inflammatory cells and a potential source of oxidative loads associated with ventilator-induced lung injury (VILI) (Figure S1). Increased neutrophil counts were observed in mice treated with bleomycin subjected to high-tidal-volume mechanical ventilation (MV) compared with the other MV treatment groups and the nonventilated control mice (Figure S2). The increase of lung inflammation in mice administered high-tidal-volume MV and treated with bleomycin were substantially reduced after the administration of AS605240 and in PI3K-γ-deficient mice (Figure S3).

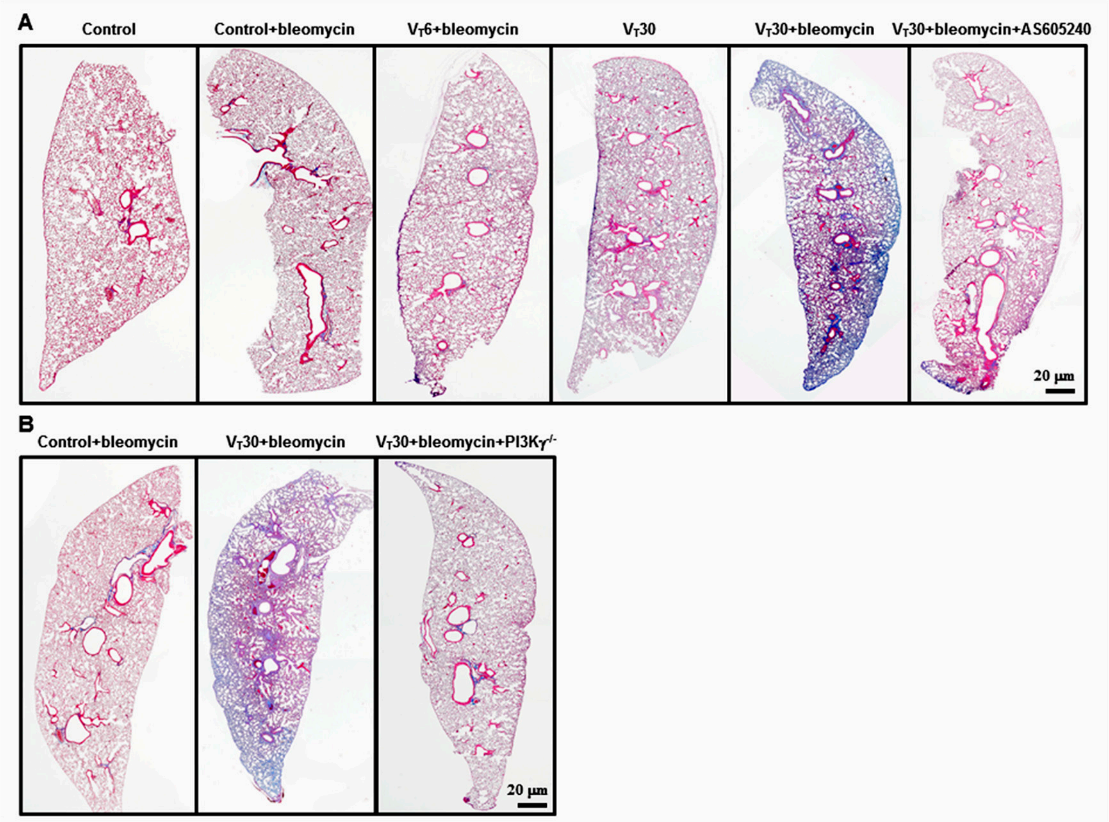

Figure S1. Reduction of lung stretch-induced collagen production by AS605240 and in PI3K-γ-deficient mice. (A, B) Representative micrographs (x40) with Masson's trichrome staining of paraffin lung sections (n = 5 per group) of lung tissue after five days of bleomycin administration were from nonventilated control mice and mice ventilated at a tidal volume of 6 mL/kg

or 30 mL/kg for 5 h with room air. AS605240 5 mg/kg was given intraperitoneally 1h before ventilation. Scale bars represent 20 μm.

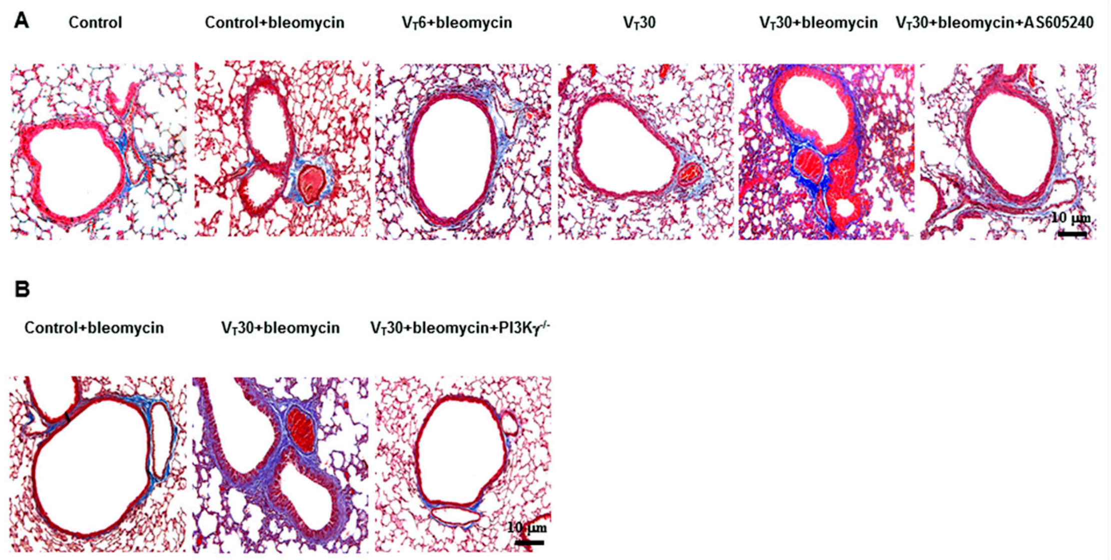

Figure S2. Reduction of lung stretch-induced collagen production by AS605240 and in PI3K-γ-deficient mice. (A, B) Representative micrographs (x200) with Masson's trichrome staining of paraffin lung sections (n = 5 per group) of lung tissue after five days of bleomycin administration were from nonventilated control mice and mice ventilated at a tidal volume of 6 mL/kg or 30 mL/kg for 5 h with room air. AS605240 5 mg/kg was given intraperitoneally 1h before ventilation. Scale bars represent 10 μm.

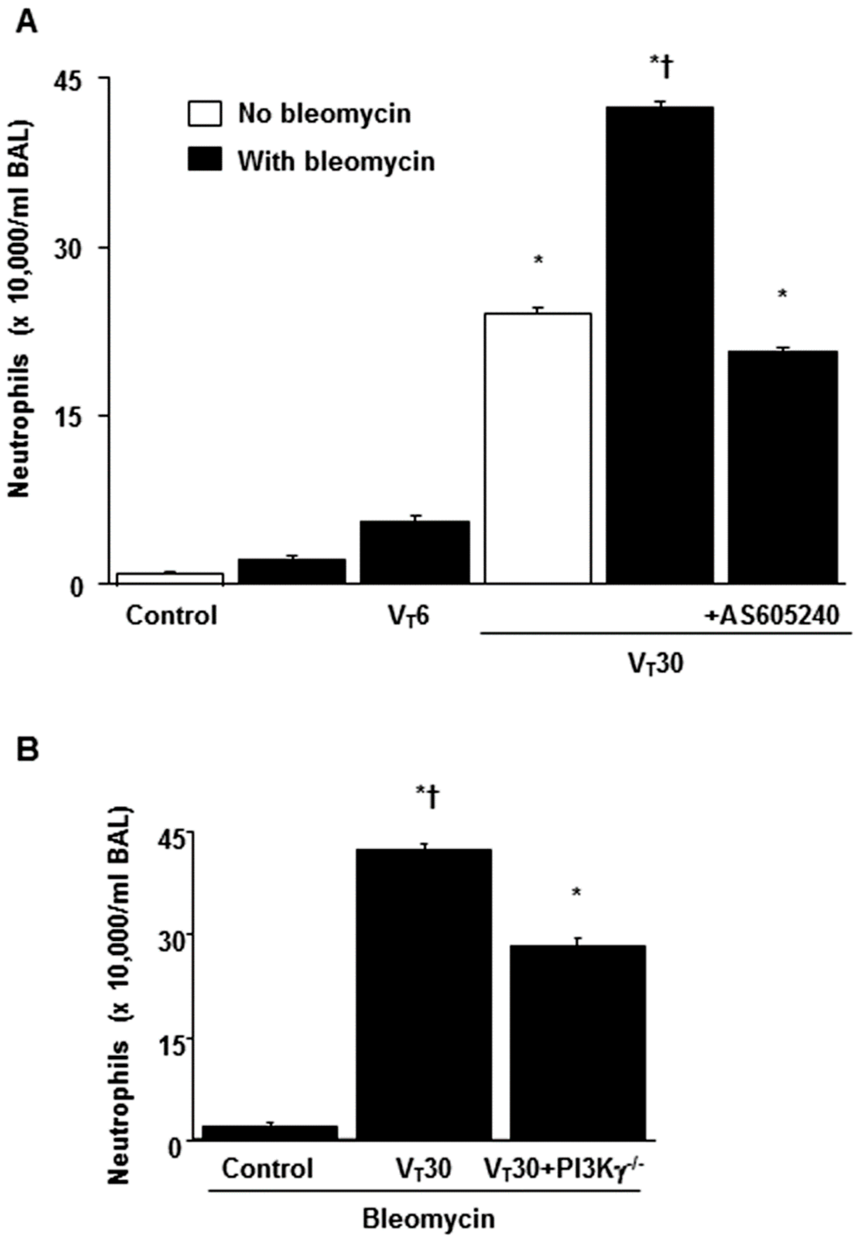

Figure S3. Inhibition of lung stretch-mediated neutrophil infiltration by AS605240 and in PI3K-γ-deficient mice. Five days after administering bleomycin, (**A, B**) **Neutrophil counts** in BAL fluid were from the lungs of nonventilated control mice and those subjected to a tidal volume at 6 mL/kg or at 30 mL/kg for 5 h with room air (n = 5 per group). AS605240 5 mg/kg was given intraperitoneally 1h before ventilation. * P< 0.05 versus the nonventilated control mice with bleomycin pretreatment; †P< 0.05 versus all other groups. BAL = bronchoalveolar lavage; PI3K-γ = phosphoinositide 3-kinase-γ.

Table S1. Physiologic conditions at the beginning and end of ventilation.

	Nonventilated	Nonventilated with bleomycin	V_T 6 ml/kg with bleomycin	V_T 30 ml/kg with bleomycin	V_T 30 ml/kg with bleomycin, AS605240	V_T 30 ml/kg with bleomycin, $PI3K\gamma^{-/-}$
PH	7.42±0.05	7.38±0.03	7.35±0.07	7.37±0.03	7.38±0.06	7.39±0.04
PaO2 (mmHg)	98.2±0.4	96.0±0.3	86.1±0.4*	76.9±2.8*	85.1±2.4*	87.4±2.1*
PaCO2 (mmHg)	39.1±0.2	39.6±0.1	40.1±1.5	36.5±1.4	37.2±1.5	37.2±1.3
MAP (mmHg)						
Start	85.6±1.4	83.7±0.5	84.6±1.4	81.9±2.9	83.7±2.3	84.1±2.1
End	85.1±0.4	80.9±0.4	80.1±2.3*	75.4±2.6*	78.9±2.2*	79.5±2.3*
PIP (mmHg)						
Start			10.3±1.1	23.6±1.2	23.5±1.4	23.7±1.3
End			12.1±1.2	28.2±1.8	26.9±1.5	27.1±1.4

At the end of the study period, we obtained data of mean arterial pressure and arterial blood gases from the nonventilated control mice and mice ventilated at a tidal volume of 6 ml/kg or 30 ml/kg for 5 h (n = 10 per group). The normovolemic statuses of mice were maintained by monitoring mean artery pressure. Data are presented as means ± SDs. * indicates that $P < 0.05$ when compared to the nonventilated control mice with bleomycin pretreatment. MAP = mean arterial pressure; $PI3K\gamma^{-/-}$ = phosphoinositide 3-kinase-γ-deficient mice; PIP = peak inspiratory pressure; V_T = tidal volume.
